# 

**Published:** 1894-09

**Authors:** 


### Society Notes.

**American Public Health Association.**—The 22nd annual meeting of the American Public Health Association will be held at Montreal, Canada, Tuesday, Wesnesday, Thursday and Friday, September 25, 26, 27, 28. The preliminary announcement is on our table. Let all interested make a note of the date, and look out for further announcements as to reduced rates of travel, etc. The officers of the Association are E. P. Lachapele, M. D., President, Montreal, Can.; Irving A. Watson, M. D., Secretary, Concord, N. H.

The following topics have been selected for consideration at this meeting:

1. The Pollution of Water-Supplies.
2. The Diposal of Garbage and Refuse.
3. Animal Diseases and Animal Food.
4. The Nomenclature of Diseases and Forms of Statistics.
5. Protective Inoculations in Infectious Diseases.
6. National Health Legislation.
7. The Cause and Prevention of Diphtheria.
8. Causes and Prevention of Infant Mortality.
9. The Restriction and Prevention of Tuberculosis.
10. Car Sanitation.

11. The Prevention of the Spread of Yellow Fever.

The Executive Committee announces the following additional subjects upon which papers are invited:

12. On the Education of the Young in the Principles of Hygiene.

13. Private Destruction of Household Garbage and Refuse.

14. Disinfection of Dwellings after Infectious Diseases.

15. Inspection of School Children with Reference to the Eyesight.

All papers presented to the Association must be either printed, type-written, or in plain handwriting, and be in the hands of the Secretary at least twenty days prior to the annual meeting, to insure their critical examination as to their fulfilling the requirements of the Association.

---

**Mississippi Valley Medical Association.**—The 20th annual meeting of the Mississippi Valley Medical Association will be held at Hot Springs, Ark., November 20, 21, 22 and 23, 1894. Reduced rates of travel will be secured for all physicians who desire to attend, and the hotel accommodations are ample. An unusually attractive program has been arranged and the meeting is expected to be of course, most interesting. It is earnestly desired that a large Texas delegation shall attend, and every inducement will be offered. The Hot Springs is numbered amongst the great natural curiosities of the world, and no man should be willing to pass the whole of his life—especially living so near as Texans do—without being able to say that at least he had "been there." (For pleasure let it be understood, and not from necessity;—many people would not like it to be known that they had "been there.")

The JOURNAL is in receipt of a very neat little pamphlet descriptive of Hot Springs, and illustrated with tinted photogravures of the principal buildings—the Springs—views, etc., containing also the list of officers of the association and committees. Dr. T. E. Holland, of Hot Springs, Chairman of the Committee of Arrangements, will take pleasure in sending a copy of this pamphlet free on application, as well as to give all information relative to the meeting. Address him as above.

---

"The committee has secured the ball room of the Kimball House, Atlanta, for the next meeting of the Tri-State Medical Society October 9th, 10th and 11th. The room has fine acoustic properties. It is shut off from all noises from the street, and is admirably adapted in every respect to the purpose." Says Dr. Frank Trester Smith, the Secretary, in a letter to us.

